# Alternative quadruplex real-time PCR reactions for detection and discrimination of *Streptococcus pneumoniae* serotypes within serogroup 6

**DOI:** 10.1128/spectrum.03045-25

**Published:** 2026-07-20

**Authors:** Samuel F. Hatchette, Nicole Paterson, Ariela Polsky, Peter Robertson, Gregory R. McCracken, Zhenyu Cheng, Jason J. LeBlanc

**Affiliations:** 1 Department of Microbiology and Immunology, Dalhousie University 3688, Halifax, Nova Scotia, Canada; 2 Department of Pathology, Dalhousie University 3688, Halifax, Nova Scotia, Canada; 3 Division of Microbiology, Department of Pathology and Laboratory Medicine, Nova Scotia Health (NSH), Halifax, Nova Scotia, Canada; 4 Department of Medicine (Infectious Diseases), Dalhousie University, Halifax, Nova Scotia, Canada; University of Heidelberg, Heidelberg, Germany

**Keywords:** *Streptococcus pneumoniae*, serotype, quadruplex, PCR, CDC

## Abstract

*Streptococcus pneumoniae* causes significant morbidity and mortality worldwide, and serotyping is important to assess the burden of disease that is vaccine preventable. For serotyping, the Centers for Disease Control and Prevention (CDC) use a series of 12 real-time multiplex PCRs (rmPCRs) performed in quadruplex reactions; however, rmPCR reaction 5 (rmPCR-5) for serotypes 6A, 6B, 6C, and 6D often failed at low DNA concentrations. This study investigated the cause of rmPCR-5 failure and provided alternative rmPCRs to resolve this issue. Quadruplex rmPCR target sequences were compared to *S. pneumoniae* reference genomes. Reactions rmPCR-5 [6ABCD, 6AB, 6BD, and 6CD] and rm-PCR-11 [37, 10F, 11BC, and 18CFBA] were compared to alternative reactions rmPCR-A1 [6ABCD, 10F, 11BC, and 18CFBA] and rmPCR-A2 [37, 6AB, 6BD, and 6CD]. All rmPCRs were tested using 10-fold serial dilutions of DNA from representative serotypes, and analytical specificity was assessed using DNA from other *S. pneumoniae* serotypes or various streptococci and Gram-positive cocci. Failure of rmPCR-5 was associated with overlapping 6ABCD and 6BD targets. Separation of these targets in the alternative rmPCRs-A1 and rmPCR-A2 allowed sensitive and specific detection and discrimination of serotypes 6A, 6B, 6C, and 6D, without impacting the detection of serotypes 10F, 11BC, 18CFBA, and 37. This study highlights the importance of rigorous author and peer-review to avoid manuscript errors and unintended consequences. By explaining what caused rmPCR-5 failure and proposing alternative reactions rmPCRs-A1 and rmPCR-A2, this study demonstrates the value of scientific collaboration to ensure molecular assays best serve the scientific community.

**IMPORTANCE:**

*Streptococcus pneumoniae* is a bacterium that can cause life-threatening infections like pneumonia and meningitis, leading to millions of deaths worldwide each year. A key feature enabling *S. pneumoniae* to cause disease is its sugar coating, allowing it to avoid the immune system. These surface sugars are the target of *S. pneumoniae* vaccines. However, vaccines only protect against some sugars and understanding which ones are on the surface of *S. pneumoniae* is called “serotyping.” The Centers for Disease Control and Prevention (CDC) have protocols that allow us to predict *S. pneumoniae* serotypes by looking at its DNA. We found errors in the CDC protocols and provided a simple solution to fix them. Ultimately, having accurate serotyping protocols allows us to know how much disease is preventable by vaccine, allows us to monitor how well vaccine are working, and helps develop new vaccines if needed.

## INTRODUCTION

Despite the current availability of antibiotics, *Streptococcus pneumoniae* (or pneumococcus) remains a significant cause of mortality and morbidity worldwide with associated diseases like community acquired pneumonia (CAP) and invasive pneumococcal diseases (IPD) (1–8). Virulence in this bacterium is predominantly attributed to its polysaccharide capsule, which helps evade immune defenses like phagocytosis and complement fixation (9). Capsule structural and antigenic properties became the focus of much research in early to mid-1900s, and over 100 serotypes have been reported to date (9). The importance of serotyping became evident following the development of pneumococcal vaccines, all of which target capsular antigens (10). Serotyping helps determine the proportion of pneumococcal disease that is potentially vaccine-preventable and helps identify candidate serotypes for future vaccine formulations.

For *S. pneumoniae* serotyping, the Quellung reaction has long been considered the gold standard (11, 12). Quellung serotyping is often used by reference laboratories for national IPD surveillance using cultured *S. pneumoniae* isolates, but this method requires live bacteria, is labor-intensive and time consuming, and results can be subjective. With the exception serotype 37 that relies solely on *tts* for capsule formation, serotype-specific capsule biosynthesis genes are located within the *cps* loci of *S. pneumoniae* (9). This knowledge served as the foundation for PCR-based serotyping (13–22). Molecular methods are fast, do not rely on live bacteria, and are amenable to automation for large batch processing. These characteristics make molecular serotyping well-suited for surveillance studies (13–22).

The most widely used protocols for molecular serotyping of *S. pneumoniae* are available on the Centers for Disease Control and Prevention (CDC)’s Streplab website (https://www.cdc.gov/strep-lab/index.html), including an end-point polymerase chain reaction (PCR) for laboratories using electrophoresis resolution, as well as real-time multiplex PCRs (rmPCRs) in triplex or quadruplex formulations, which can detect 35 and 64 serotypes, respectively (20–22). These rmPCR methods have been used on DNA extracted from cultured isolates obtained from CAP and IPD surveillance (5–8, 21, 22). Due to the increased sensitivity of real-time PCR compared to conventional PCRs, the triplex rmPCRs (21) have been previously used in our laboratory for direct testing on nasopharyngeal swabs to monitor *S. pneumoniae* carriage (17–20). With past experiences with molecular serotyping for *S. pneumoniae* (17–20), our laboratory attempted to validate the CDC quadruplex rmPCRs for the same purpose. However, during validation, the quadruplex rmPCR reaction 5 (rmPCR-5) for the detection and discrimination of serotypes within serogroup 6 (i.e., serotypes 6A, 6B, 6C, and 6D) failed quality control at low concentrations of DNA. Reaction rmPCR-5 combines two PCR targets previously described in the triplex rmPCRs (21) to detect serogroup 6 (i.e., 6ABCD) and serotypes 6C and 6D (6CD), as well as two new reactions designed to detect and differentiate serotypes 6A and 6B (6AB) as well as serotypes 6B and 6D (6BD). The current study not only describes the possible reasons for rmPCR-5 failure but also proposes alternative reactions (rmPCR-A1 and rmPCR-A2) that allowed for accurate detection and discrimination of serotypes 6A, 6B, 6C, and 6D.

## MATERIALS AND METHODS

### Bioinformatic analyses

Each quadruplex rmPCR target was queried against reference genomes for each *S. pneumoniae* serotype (23) and their sequence retrieved from the National Center for Biotechnology Information (NCBI) Genbank database (Table S1) for pairwise alignment with concatenated primer and probe sequences using the NCBI Basic Local Alignment Search Tool (BLAST) (https://blast.ncbi.nlm.nih.gov/Blast.cgi). If queries based on the annotated target were not found, the concatenated sequence of the primers and probes were subjected to a standard nucleotide BLAST analysis to find the most probable target.

### Bacterial culture

Isolates of *S. pneumoniae*, other streptococci, and Gram-positive cocci (Table S2) were obtained from the −80°C biorepositories in the Division of Microbiology, Department of Pathology and Laboratory Medicine, Nova Scotia Health (NSH), the University of Manitoba (Winnipeg, MB), the Streptococci Reference Laboratory at the National Microbiology Laboratory (NML) (Winnipeg, MB), CDC Global Pneumococcal Strain Bank (Atlanta GA) or from the American Type Culture Collection (ATCC) via Cedarlane Corporation (Burlington, ON). *S. pneumoniae* and most other streptococci isolates were cultured onto Becton Dickinson and Co. (BD) Diagnostics (Mississauga, ON) Trypticase soy agar (TSA) II with 5% sheep blood and incubated at 35°C plus 5% CO_2_ overnight, except *Abiotrophia defectiva* and *Granulicatella adjacens* which were cultured on BD Chocolate agar under the same conditions. After incubation, bacteria were harvested and placed into PCR-grade water for subsequent nucleic acid extraction or were archived at −80°C in CryoStar Microbiological Preservative vials (Innovatek Medical Inc., Vancouver, BC) as recommended by the manufacturer.

### Nucleic acid extraction

Bacterial stock volumes of 200 µL (or 10-fold serial dilutions of these bacterial suspensions in PCR-grade water) were subjected to combined DNA and RNA (i.e., total nucleic acids [TNA]) extraction using a MagNA Pure 96 DNA and viral RNA small volume kit on a MagNA Pure 96 instrument (Roche Diagnostics GmbH, Mannheim, Germany), following manufacturer recommendations. TNAs were eluted in a final volume of 50 µL, with 5 µL used as template for all real-time PCR amplifications.

### Real-time multiplex PCR

Each rm PCR was performed using either the PerfeCTa qPCR Toughmix (Quantabio, Radnor, PA) (22) or QuantiTect Multiplex PCR No ROX (NR) Kit (Qiagen GmbH, Hilden, Germany), following manufacturer instructions. All rmPCRs were performed in 25 µL reactions consisting of 5 µL of template TNA extracted from bacterial cultures (or dilutions), 1× of enzyme mix/buffer, and primer and probe concentrations reported by Velusamy et al. (22). The quadruplex rmPCR combinations are listed in Table S1. Primers and dual-labeled probes were synthesized by Sigma Genosys (Oakville, ON) and Integrated DNA Technologies (Coralville, IA), respectively. PCR amplifications were performed using a Life Technologies Applied BioSystems 7500 Fast instrument for both PCR reagent types; however, thermocycling conditions differed slightly. For CDC quadruplex rmPCRs using the PerfeCTa Toughmix kit, thermocycling conditions consisted of an initial activation of the DNA polymerase at 95°C for 10 min, followed by 40 cycles of denaturation at 95°C for 15s and a combined annealing/extension step at 60°C for 60s. For rmPCRs using the QuantiTect kit, thermocycling included an initial activation at 95°C for 15 min, followed by 40 cycles of denaturation at 95°C for 45s and a combined annealing/extension step at 60°C for 75s. Fluorescence emission was captured at the extension stage in the FAM, VIC, ROX, and Cy5 channels, and threshold cycle (Ct) values were determined by the manufacturer’s software. Results showing exponential amplification and Ct value less than 37 were considered positive.

Prior to methods comparisons between the monoplex and traditional quadruplex or alternative quadruplex rmPCRs, DNA from the targeted *S. pneumoniae* serotypes (obtained from TNA extraction) were normalized based on Ct values for a common genomic target, autolysin (*lytA*). Briefly, a real-time PCR targeting *lytA* (18) was performed and TNAs were normalized by dilution in PCR-grade water to attain Ct values of 30 (±2) to represent specimens with a low concentration of *S. pneumoniae* TNA and Cts of 24 (±2) to represent specimens with high bacterial loads.

Real-time reactions were performed as described for the CDC quadruplex rmPCR reactions (22) with two modifications: (i) the forward primers for serotypes 14 and 19A were extended on the 5′end by 5 and 7 bp, respectively, to meet melting temperature (Tm) requirements for reproducible detection and (ii) all VIC fluorophores were changed to MAX. Monoplex PCRs were also conducted for each target within the quadruplex rmPCRs or as alternative rmPCRs with target permutations. Traditional quadruplex rmPCRs (22) included rmPCR-5 (for detection and discrimination of serotypes 6ABCD, 6BD, 6AB, 6CD) and rmPCR-11 (for serotypes 37, 11B/C, 10F, 18CFBA) (Table S1).

### Amplicon analysis using an automated electrophoresis

Following the failure of quadruplex rmPCR-5, electrophoresis was performed to investigate the amplicons produced during amplification reactions. Monoplex PCRs using individual primer probes for each target were used as comparators for the traditional and alternative quadruplex PCRs. For electrophoresis, amplicons were diluted 1:50 in PCR-grade water to avoid any buffer interference with the electrophoresis. Then, 2 µL of the diluted amplicons (or DNA ladder) was mixed with an equal volume of Agilent High Sensitivity D1000 Sample Buffer (Agilent, Santa Clara, CA) and loaded onto an Agilent TapeStation 4150. Electrophoresis was conducted using an Agilent High Sensitivity D1000 ScreenTape following the manufacturer instructions. Each run contained up to 14 specimens with 2 DNA ladders. Amplicon sizes were analyzed by the manufacturer software, and each lane was cropped at the upper marker (1,500 base pair, bp) and lower markers (25 bp). For consistency in data presentation between runs, the spacing of the ladder was used as a reference for sizes.

### Validation of the alternative quadruplex rmPCRs A1 and A2

The alternative rmPCRs were a permutation of rmPCR-5 and rmPCR-11. Alternative rmPCR reaction 1 (rmPCR-A1) consisting of serotypes 6ABCD, 11B/C, 10F, and 18CFBA and alternative rmPCR reaction 2 (rmPCR-A2) grouped targets for serotypes 37, 6BD, 6AB, and 6CD. The rationale for this permutation was a separation of the 6ABCD target from 6BD which targets overlapping regions of *wciP* while maintained the same number of detectable serotypes.

Traditional rmPCR-5 and rmPCR-11 and alternative quadruplex rmPCRs rmPCR-A1 and rmPCR-A2 were compared using 10-fold serial dilutions in PCR-grade water of normalized *S. pneumoniae* DNA from representative serotypes 6A, 6B, 6C, 6D, 10F, 11B, 18C, and 37. For each detectable serotype, comparative plots between traditional and alternative quadruplex rmPCRs were presented following Probit analysis (https://biostats.shinyapps.io/LOD_probit/) to estimate the limit of detection (LoD) at a 95% probability including confidence intervals (CI) (24–26). The LoD of each serotype detection was expressed as log_10_ copies/mL.

To assess specificity, various temporally and geographically diverse *S. pneumoniae* isolates, previously characterized by Quellung serotyping, were tested individually. These included serotypes 6A (*n* = 18), 6B (*n* = 19), 6C (*n* = 20), 6D (*n* = 15), 10F (*n* = 10), 11B (*n* = 10), 11C (*n* = 10), 18A (*n* = 12), 18B (*n* = 20), 18C (*n* = 20), 18F (*n* = 15), and 37 (*n* = 10), as well as 80 *S. pneumoniae* serotypes not detected using these reactions (1, 2, 3, 4, 5, 7A, 7B, 7C, 7F, 8, 9A, 9L, 9N, 9V, 10A, 10B, 10C, 11A, 11D, 11E, 11F, 12A, 12B, 12F, 13, 14, 15A, 15B, 15C, 15F, 16A, 16F, 17A, 17F, 19A, 19B, 19C, 19F, 20, 21, 22A, 22F, 23A, 23B, 23F, 24A, 24B, 24F, 25A, 25F, 27, 28A, 28F, 31, 32A, 32F, 33A, 33B, 33C, 33D, 33F, 34, 35A, 35B, 35C, 35F, 36, 37, 38, 39, 40, 41A, 41F, 42, 43, 44, 45, 46, 47, and 48). The rmPCR-A1 and rmPCR-A2 reactions were tested for specificity using panels of pneumococcal serotypes listed above and with various streptococci and Gram-positive cocci (Table S2).

## RESULTS

While accurate detection was seen for most serotypes during validation of the CDC quadruplex rmPCRs (Table S1), all targets from rmPCR-5 targeting the detection and discrimination of serotypes within serogroup 6 (i.e., serotypes 6A, 6B, 6C, and 6D) failed quality control at low concentrations of DNA but were detected at high DNA concentrations (Fig. S1 and S2). The cause of failure was investigated using bioinformatic analyses, amplicon electrophoresis, comparisons between monoplex targets, and the traditional quadruplex rmPCR. To mitigate the serogroup 6 quadruplex rmPCR detection failures, two alternative quadruplex reactions (rmPCR-A1 and rmPCR-A2) were validated against the traditional rmPCRs. Analytical sensitivity analysis using 10-fold serial dilutions with normalized serotype 6A, 6B, 6C, and 6D DNA and specificity analyses with clinical isolates and reference strains *S. pneumoniae* and related organisms.

### Bioinformatic analyses revealed target site mis-annotations and serogroup 6 PCR target overlap

Bioinformatic analyses were first used to map each quadruplex PCR target site (delineated by the forward and reverse primers) against *S. pneumoniae* reference genomes for representative serotypes. During verification of these serotype-specific target sites, several inconsistencies were noted, including typos and gene mis-annotations (Table S1). The Velusamy et al. (22) manuscript itself presents a summary table for serotypes targeted by each quadruplex rmPCR in which reaction 8 includes serotype 32; however, bioinformatic analyses and laboratory testing confirmed the primers and probe detect serotype 34 (which was correctly annotated in the oligonucleotide list). In the supplemental table describing the primer/probe sequences (22) and posted on the CDC StrepLab website, there were six rmPCR target sites that were annotated incorrectly (Table S1). For example, the target site for serotypes 9N/L was labeled as *wzy* but bioinformatic analyses revealed the primers and probes bind *wzx*. Similarly, the target site for serotypes 7F/A was annotated as *wzy* but was found to target *wcwH* instead. During validation, accurate detection of serotypes 9N, 9L, 7F, and 7A was confirmed despite these mis-annotations; therefore, these had little consequence.

**Fig 1:**
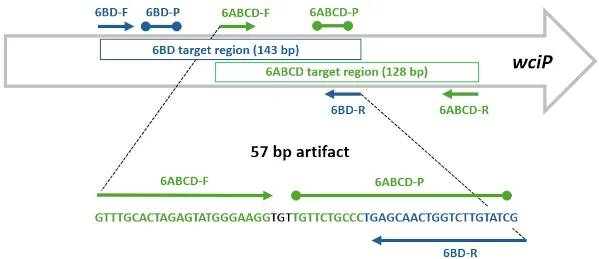
Possible artifact generation from 6ABCD and 6BD PCR target overlap. The rmPCR targets 6ABCD and 6BD are located within *wciP* (large gray arrow). The 143 bp 6BD target region (in blue) is flanked by its forward and reverse primers (6BD-F and 6BD-R, respectively; illustrated as small blue arrows) and the probe (6BD-P) is located within this region. Similarly, the 128 bp fragment for the 6ABCD target (in green) is flanked by small green arrows corresponding to its forward (6ABCD-F) and reverse (6ABCD-R) primers, and internal to this region is the probe binding site (6ABCD-P). Given both 6ABCD and 6BD targets overlap, the 6ABCD-F and 6BD-R primers could generate artifacts. These included among others a 57 bp artifact whose sequence is depicted (derived from the 6ABCD-F and 6BD-R primers), a 214 bp artifact (derived from the 6BD-F and 6ABCD-R primers), and a 155 bp product (from the combination of the 6ABCD-R primer and extension of the 6BD-P probe). Of note, the 6BD-R primer is complimentary to a region within the 6ABCD-P that could also impact 6ABCD target detection.

On the other hand, targets within rmPCR-5 for detection and discrimination of serotypes within serogroup 6 were all mis-annotated. The primers and probes for the 6AB target aligned to an 89 bp region of *wciN*α (also known as *wciN*), not *wciP* as annotated in the manuscript. The 6CD target site was shown to be a 149 bp segment of *wciN*β, not *wciN*. The 6ABCD target site was incorrectly annotated as *wzy* but revealed to be a 128 bp region of *wciP*. Interestingly, like 6ABCD, the 143 bp of the 6BD target site was also *wciP*, not *wciN*β as annotated. The most concerning observation was the possible artifact generation from the overlapping *wciP* sequences of the 6ABCD and 6BD targets. Possible PCR artifacts explained by the overlapping *wciP* targets include a 57-bp fragment from the forward primer of the 6ABCD target (6ABCD-F) and the reverse sequence of the 6BD target (6BD-F), a 214 bp fragment generated from the 6BD forward (6AB-F) and reverse 6ABCD (6ABCD-R) primers, and a 155 bp product from a combination of the 6ABCD-R primer and extension of the 6BD-P probe (Fig. 1). It should be noted that the 6BD-R primer sequence almost completely overlaps with the 6ABCD-P probe binding site (Fig. 1).

### Comparison of monoplex and quadruplex real-time PCRs and amplicon analyses

To understand the reasons for the failure of quadruplex rmPCR-5, monoplex real-time PCRs for each individual PCR target (6ABCD, 6AB, 6BD, and 6CD) were compared to those of the quadruplex rmPCR-5 using low and high concentrations of DNA extracted from serotypes 6A, 6B, 6C, and 6D (Fig. S1 and S2). Following automated electrophoresis, all monoplex PCRs showed amplicons of expected sizes: the 128 bp 6ABCD (*wciP*) target was present in each serotypes tested (6A, 6B, 6C, and 6D); the 89 bp 6AB (*wciN*α) target was present for serotypes 6A and 6B but absent for 6C and 6D; the 143 bp 6BD (*wciP*) target was seen in serotypes 6B and 6D but is absent in serotypes 6A and 6C; and the 149 bp 6CD (*wciN*β) target was seen for serotypes 6C and 6D but absent for 6A and 6B. While each individual serogroup 6 target was amplified as expected in the monoplex real-time PCRs, the quadruplex rmPCRs did not show any of expected amplicons for any of the serotypes tested, and many primer dimers and artifact bands were visible (Fig. S2).

### Validation of the alternative quadruplex rmPCRs A1 and A2

Primers and probes for the 6ABCD (*wciP*) target of rmPCR-5 were switched with those for serotype 37 (rmPCR-11), as both were detected in the FAM-fluorescence channel. The alternative quadruplex rmPCR-A1 grouped primers and probes for the 6ABCD target to those targeting serotypes 11BC, 18CFBA, and 10F, and the second alternative quadruplex rmPCR-A2 grouped targets for serotypes 37, and those to resolve serogroup 6 (6AB, 6BD, and 6CD). This simple solution maintained the same number of detectable targets, but the separation of the 6ABCD and 6BD targets allowed accurate and sensitive detection and discrimination of serotypes 6A, 6B, 6C, 6D, 10F, 11B/C, 18C/F/B/A, and 37.

**Fig 2:**
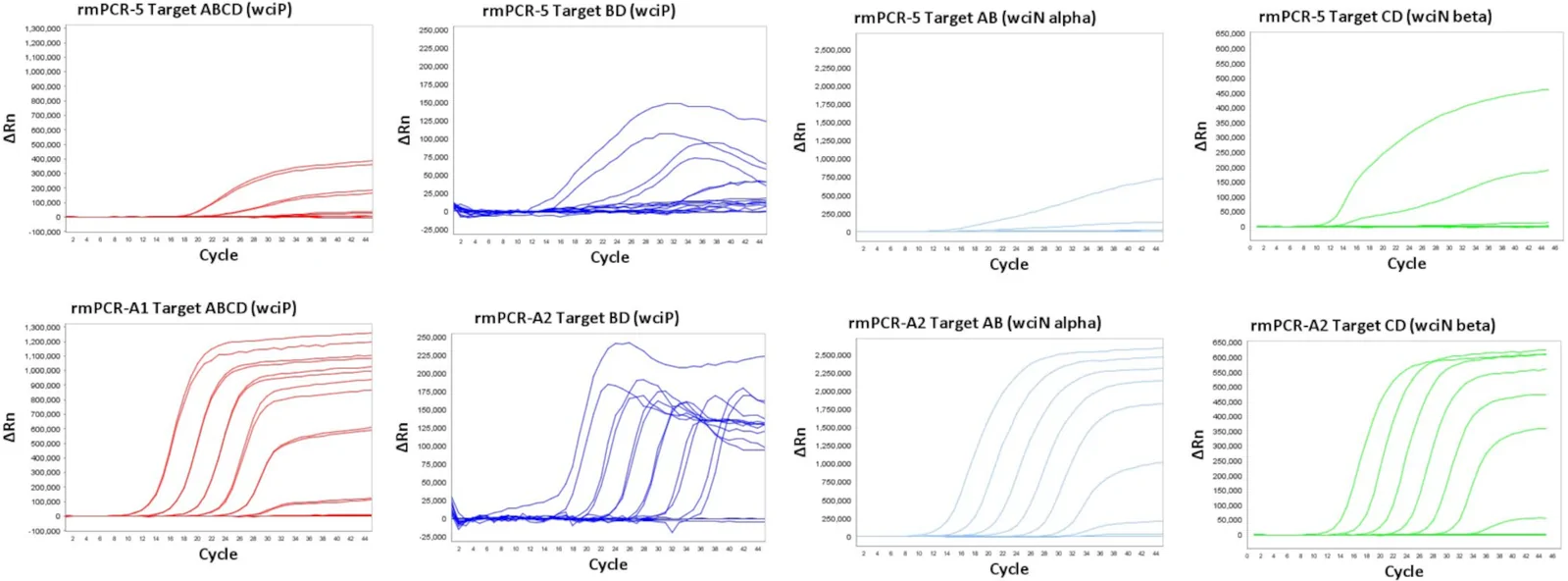
Representative amplification plots comparing traditional and alternative rmPCRs. Displayed are representative amplification plots for 10-fold serial dilutions of normalized DNA from serotypes 6B and 6D for all rmPCR-5 targets compared the same targets in the rmPCR-A1 and rmPCR-A2 reactions. Anticipated results are amplification of both targets in the 6ABCD (*wciP*) and 6BD (*wciP*) targets, amplification of a single target for serotype 6B for target 6AB (*wciNα*), and amplification of a single target for serotype 6D for target 6CD (*wciNβ*). Of note, serotypes 6A and 6C (not shown) have similar curves for all reactions for the 6ABCD target but are negative for the 6BD target. Serotype 6A amplification occurs identically to serotype 6B for the 6AB target and is negative for the 6CD target. Serotype 6C amplification occurs identically to serotype 6D for the 6CD target and is negative for the 6AB target. The sum of these reactions allows accurate discrimination of serotypes 6A, 6B, 6C, and 6D using rmPCR-A1 and rmPCR-A2, but the targets fail with rmPCR-5 unless high DNA concentrations are used.

**Fig 3:**
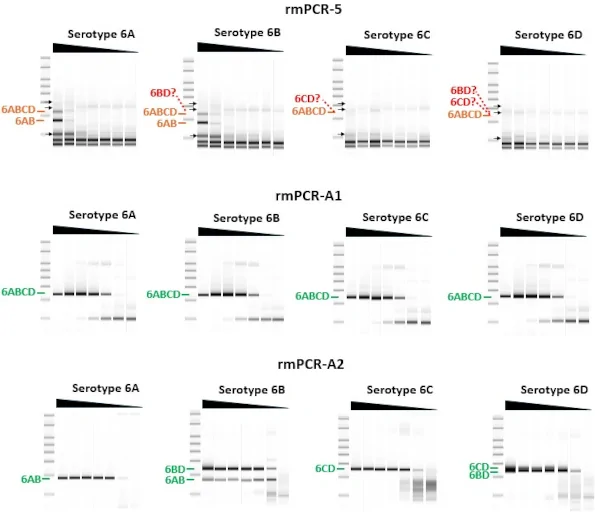
Analyses of amplicons generated from rmPCR-5 and the alternative reactions rmPCR-A1 and rmPCR-A2. Displayed are representative amplicons produced from 10-fold serial dilutions of normalized DNA from *S. pneumoniae* serotypes 6A, 6B, 6C, and 6D for all targets in the traditions rmPCR-5 reactions compared the same targets in the alternative reactions rmPCR-A1 and rmPCR-A2. The expected product sizes for the rmPCR targets are as follows: 128 bp for 6ABCD (*wciP*); 143 bp for 6BD (*wciP*); 89 bp for 6AB (*wciN*α); and 149 bp target for 6CD (*wciN*β). Target that passed validation is labeled in a green font, an orange font signifies the detection was only at high DNA concentrations, and the red font indicates a target that where detection was absent or weak. At low concentrations of *S. pneumoniae* DNA, the rmPCR-5 quadruplex reaction for serotypes 6A, 6B, 6C, and 6D often failed detection for anticipated targets and artifacts were generated with predicted sizes of 57 bp, 155 bp, and 214 bp (see black arrows).

Interestingly following 10-fold serial dilutions of *S. pneumoniae* DNA for serotype 6A, 6B, 6C, and 6D, detection failure for rmPCR-5 targets was not seen at high DNA concentrations but was evident at lower DNA concentrations (Fig. 2), consistent with our initial validation data comparing rmPCR-5 to monoplex PCRs for each target (Fig. S1 and S2). Apart from the obvious lack of sensitivity with the rmPCR-5 reaction targets for all serotypes, the amplification profiles for serotypes 6A, 6B, 6C, and 6D (when detected) were often aberrant, unlike the typical sigmoidal curves and large dynamic range seen with rmPCR-A1 and rmPCR-A2 reactions (Fig. 2). Following electrophoresis of the amplicons produced from each reaction, the lack of sensitivity of rmPCR-5 was again evident compared to targets detected in the rmPCR-A1 and rmPCR-A2 reactions (Fig. 3). Interestingly, artifacts were prominent in rmPCR-5 reactions, particularly at low DNA concentration (Fig. 3). These included among others the predicted 57 bp artifact (derived from the 6ABCD-F and 6BD-R primers), a 214 bp artifact (derived from the 6BD-F and 6ABCD-R primers), and a 155 bp product from the combination of the 6ABCD-R primer and extension of the 6BD-P probe (Fig. 1 and 3). In the traditional quadruplex rmPCR-5, there was also a high concentration of primer dimers (Fig. 3). Primer dimers were less evident when using the rmPCR-A1 and rmPCR-A2 reactions, and non-specific artifacts had no impact of serotype detection.

**Fig 4:**
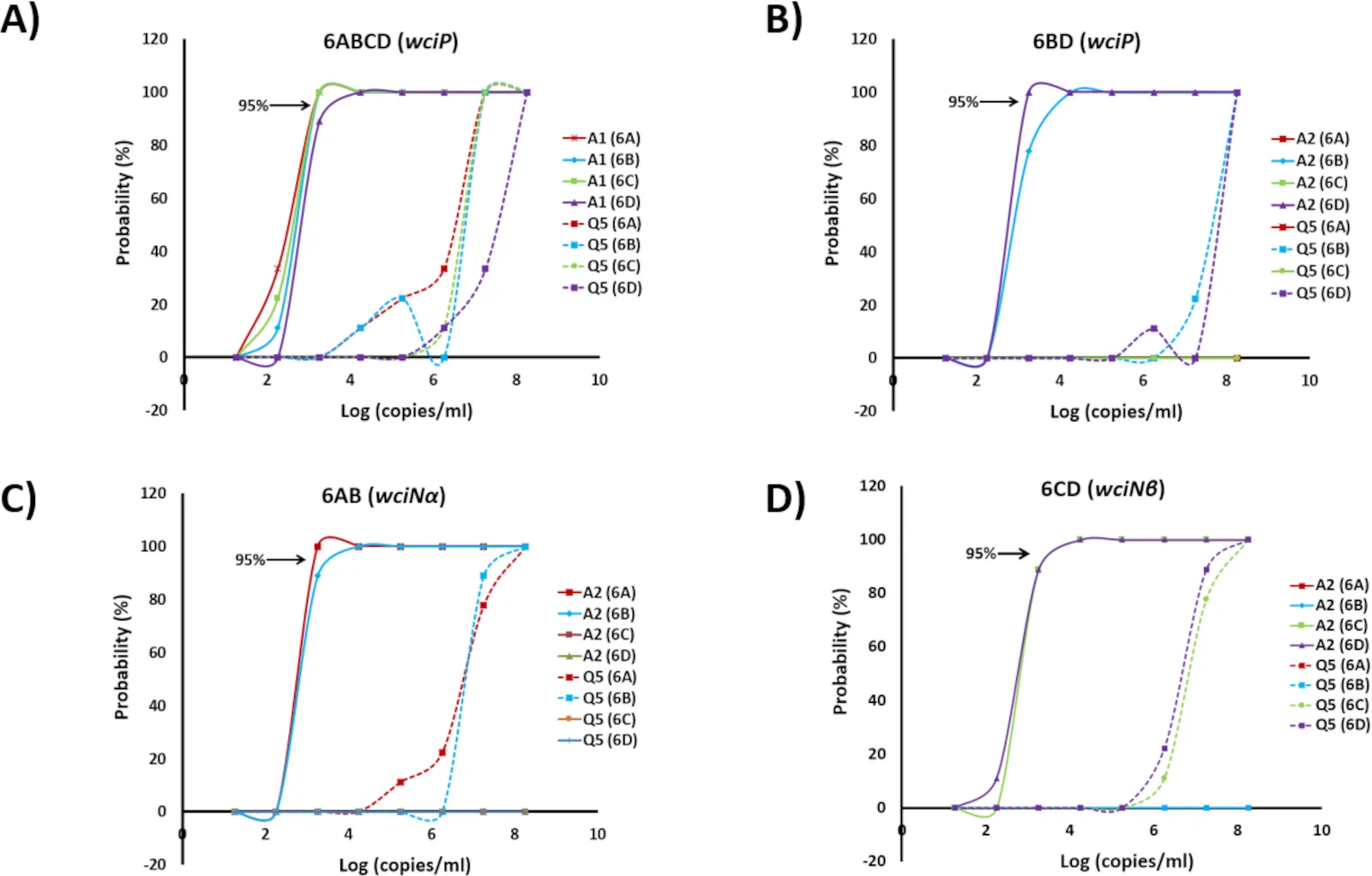
Probit analysis illustrating the concentration-dependent performance of traditional and alternative quadruplex rmPCRs. Ten-fold serial dilutions (*n* = 9) were performed with normalized DNA from *S. pneumoniae* for serotypes 6A, 6B, 6C, and 6D, and each dilution was subjected to the traditional quadruplex rmPCR-5 (denoted Q5) or the alternative quadruplex rmPCR-A1 and rmPCR-A2 (denoted A1 or A2) to identify the following targets: (**A**) 6ABCD (*wciP*); (**B**) 6BD (*wciP*); (**C**) 6AB (*wciNα*); and 6CD (*wciNβ*). Results are shown with dashed lines for rmPCR-5 and with solid lines for the alternative reactions rmPCR-A1 and rmPCR-A2. Shown in panels **A–D** are serotypes 6A (red), 6B (blue), 6C (green), 6D (purple).

Probit analyses on DNA from each detectable serotype showed rmPCR-A1 and rmPCR-A2 were 4- to 5-log more sensitive than the traditional rmPCR-5 reaction for serotypes 6A, 6B, 6C, and 6D (Fig. 4), and the sensitivity remained unchanged for serotypes 10F, 11BC, 18ABCF, and 37 (Fig. S3; Table 1). No cross reactions were seen with other *S. pneumoniae* serotypes, streptococci, or Gram-positive cocci. Reactions rmPCR-A1 and rm-PCR-A2 were able to detect all geographically and temporally diverse strains of targeted serotypes (data not shown).

**TABLE 1:** Analytical sensitivity comparison between traditional and alternative quadruplex reactions^*a*^

Serotype	Target	Traditional quadruplex		Alternative quadruplex	
		Reaction	LoD in log copies/mL (95% CI)	Reaction	LoD in log copies/mL (95% CI)
6A	ABCD (*wciP*)	5	7.908 (6.935 to 8.882)	A1	3.258 (2.631 to 3.885)
6B			8.298 (7.251 to 9.345)		3.234 (2.726 to 3.743)
6C			7.234 (6.726 to 7.743)		3.266 (2.679 to 3.853)
6D			8.502 (7.673 to 9.331)		3.470 (3.016 to 3.925)
6A	BD (*wciP*)	5	ND	A2	ND
6B			8.226 (7.668 to 8.844)		3.720 (3.147 to 4.293)
6C			ND		ND
6D			8.752 (7.873 to 9.631)		3.234 (2.821 to 3.648)
6A	AB (*wciNα*)	5	8.184 (7.279 to 9.088)	A2	3.234 (2.801 to 3.648)
6B			8.298 (7.251 to 9.345)		3.470 (3.016 to 3.925)
6C			ND		ND
6D			ND		ND
6A	CD (*wciNβ*)	5	ND	A2	ND
6B			ND		ND
6C			7.894 (7.199 to 8.588)		3.266 (2.679 to 3.853)
6D			7.676 (6.984 to 8.368)		3.470 (3.016 to 3.925)
10F	*wcrC*	11	3.616 (2.988 to 4.243)	A1	3.266 (2.679 to 3.853)
11B	*wzy*	11	3.080 (2.439 to 3.721)	A1	2.932 (2.304 to 3.560)
18C	*wzy*	11	3.258 (2.631 to 3.885)	A1	3.266 (2.679 to 3.853)
37	*tts*	11	3.258 (2.631 to 3.885)	A2	3.188 (2.548 to 3.828)

^*a*^ Confidence intervals (CI); limit of detection (LoD); not detected (ND).

## DISCUSSION

Accurate serotyping methods are important to assess the current epidemiology of circulating *S. pneumoniae* and to help policy makers assess the burden of illness possibly preventable by pneumococcus vaccines. The CDC protocols for multiplex PCR-based serotyping are valuable surveillance tools that are widely used in pneumococcal disease surveillance worldwide. However, this study showed one of CDC quadruplex rmPCRs failed quality control during validation in our laboratory. The cause was investigated and determined to be due to overlapping PCR targets in reactions targeting 6ABCD and 6BD. To circumvent this error in PCR design, two alternative quadruplex reactions rmPCR-A1 and rmPCR-A2 were validated using a simple target permutation from the traditional quadruplex rm-PCR-5 and rmPCR-11 reactions.

Failures with multiplex PCRs can arise from various factors. In this study, these include primer or probe interactions, reagent competition, artifact formation, and possibly other factors from using different reagents and thermocycler compared to the traditional quadruplex PCRs. As the detection failure only occurred for serotypes within serogroup 6 detected with rmPCR-5, this would suggest the reagents and instrument were not the likely culprit of the target detection failures. While artifacts are possible for all rmPCRs, those predicted from the *wciP* target overlap in the rmPCR-5 reaction were clearly visible along with a high amount of primer dimers, which suggested a problem with the rmPCR-5 reaction. Interestingly, serial dilution of serotype 6A, 6B, 6C, and 6D DNA and Probit analyses showed target detection failure with rmPCR-5 was not seen at high *S. pneumoniae* DNA concentrations, but frequent at lower concentrations. This observation could potentially explain why the CDC did not report any performance issues for the serogroup 6 quadruplex. Testing at the CDC relies mostly on *S. pneumoniae* isolates from IPD surveillance programs where the bacterial DNA load would be high and less prone to failure. However, failure of the quadruplex rmPCR-5 reaction would be more common if applied to clinical specimens, as low DNA concentrations would be more frequent and could potentially lead to underestimation of pneumococcal carriage or disease burden.

The cause of the rmPCR-5 failure became evident following bioinformatic analyses that showed overlapping targets for 6ABCD and 6BD within *wciP*. These genes were annotated incorrectly by Velusamy et al. (22) and on the pneumococcal serotyping primer/probe list available on the CDC Streplab website at the time of this study. The 6BD target was a novel design (22), but interestingly, the 6ABCD target was correctly annotated as *wciP* when used in a previously published triplex rmPCR reaction (21). Other target site mis-annotations were observed in the same manuscript but are less concerning as they would have no impact on result interpretations (Table S1). These include the other serogroup 6 quadruplex PCR targets 6AB and 6CD, as well as the gene targets for serotypes 7F/A and 9N/L. Ideally, all information in peer-reviewed scientific manuscripts would be flawless, but manuscripts are often imperfect and contain minor typographical or grammatical errors that are ignored. While some allowance for error must be made, typos could lead to result misinterpretation or experiment failure (27, 28). Examples in previous publications include a primer duplication when describing the forward and reverse primers for a *S. pneumoniae* real-time PCR detection of serotypes 11A/D (28) and an incorrect genome data entry in a public database that was used as a reference strain by many publications (17, 23). Despite the possibility of deleterious outcomes when erroneous, the accuracy of gene annotations or sequences for primers, probes, or reference genomes are often overlooked during peer-review (27–29). There is a growing area of interest in the use of artificial intelligence to spot errors in research publications, and this might become more useful as the technology evolves (29). For now, errors that could have an impact on study findings or method reproducibility should be reported. Publication retraction might be warranted with major errors (e.g., plagiarism, bias, or fraud), but a simple corrigendum might be appropriate for both the Velusamy et al. manuscript (22) and any related information using these data (e.g., CDC Streplab website) given that simple edits could remedy the errors. An addendum could also be considered with a description of the alternative quadruplex rmPCRs and their rationale (27, 29).

The goal of this study was not to simply explain the design error that caused irreproducible results for serogroup 6 to individuals using rmPCR-5 for surveillance, but to provide a simple solution. A full redesign of primer and probe sequences was unnecessary as reactions worked with the monoplex real-time PCRs. Instead, this study separated the detection of 6ABCD and 6BD using target permutation in two alternative quadruplex reactions rmPCR-A1 and rmPCR-A2. This strategy allowed the detection and discrimination of serotypes 6A, 6B, 6C, and 6D and retained the sensitive and specific detection of serotypes 10F, 11B, 18C, and 37.

During the timeframe of peer-review for this manuscript, its findings were communicated with the CDC and presented at a national conference in Canada. Recently, the CDC has updated the Streplab pneumococcal primer and probes list to correct the serogroup 6 rmPCR target mis-annotations and have taken additional step to prevent rmPCR-5 failures. These include rmPCR target permutations and use of the internal quenchers paired with 6-carbon spacers on the probe 3′end to prevent probe extension by the DNA polymerase during amplification. Like suggested in this manuscript, the 6ABCD and 6BD targets are now separated in the updated CDC primer/probe list and accompanying table describing the serotypes within the revised rmPCRs. The targets for 6ABCD and 6CD are now tested along with those for serotypes 33F/33A/37 and 11A/11D/11E in a revised rmPCR-4 reaction, whereas the targets 6AB and 6BD are now found in a revised rm-PCR-5 with targets for serotypes 20 and 23B. This 2-target permutation of serotypes detected by rmPCR-4 and rmPCR-5 is logical and based on IPD serotype epidemiology in North America. Of note, the alternative reactions rmPCR-A1 and rmPCR-A2 in this manuscript were chosen not only to separate 6ABCD from 6BD to prevent target detection failures, but the rmPCR-A2 would only be required for rmPCRs testing positive for targets 6ABCD or 33F/33A/37, as rmPCR-A2 allows discrimination of serotypes 6A, 6B, 6C, and 6D as well as detects serotype 37. In large surveillance studies, this proposed algorithm reflects costs and time savings by reducing the number of rmPCRs required for serotyping.

It should be noted that this study is not without limitations. First, this study did not have access to a direct comparison of the alternative quadruplex rmPCRs with the CDC. The instrument used by Velusamy et al. (22) (i.e., Stratagene Mx3005P) could not be purchased as it has been discontinued; therefore, the quadruplex rmPCRs were validated on another thermocycler (i.e., Applied Biosystems 7500 Fast) using common commercially available reagents used by the CDC (i.e., PerfeCTa Toughmix). Similar results were obtained for all experiments when tested with another PCR kit commonly used in clinical or public health laboratories (i.e., QuantiTect No ROX Multiplex PCR), but the data were not presented. Next, while this study demonstrated target detect failure for the traditional rmPCR-5 reaction at low concentrations of *S. pneumoniae* DNA, this phenomenon was not observed at high DNA concentrations. It could be argued that this is a 4- to 5-log reduction in sensitivity rather than a complete rmPCR-5 reaction failure (Table 1). However, given the extent of the analytical sensitivity reduction, irreproducible results and aberrant amplification curves that sometimes occur even at high DNA concentrations, the traditional rmPCR-5 results were deemed a validation failure.

Accurate and reproducible serotyping methods are needed to assess the burden of vaccine-preventable illness and to monitor current pneumococcal disease epidemiology. While errors during manuscript preparation are often caught during peer review, others can remain and be published. As seen in this study, errors can have unintended consequences. Authors should be vigilant when preparing manuscripts and more rigorous quality control checks should be enforced during peer-review. For protocol users, validation should always occur prior to test implementation. Fortunately, the validation data provided in the study demonstrated that the alternative quadruplex reactions rmPCR-A1 and rmPCR-A2 were a simple and sensitive solution for accurate results for serotypes 6A, 6B, 6C, and 6D. The recent revisions of the CDC rmPCR reactions 4 and 5 should also be a suitable solution, but no validation have been published to date.

## Data Availability

Available data can be found in supplemental material.

## SUPPLEMENTAL MATERIAL

Supplemental Material spectrum.03045-25-s0001.pdf Fig. S1 to S3; Tables S1 and S2.

ASM does not own the copyrights to Supplemental Material that may be linked to, or accessed through, an article. The authors have granted ASM a non-exclusive, world-wide license to publish the Supplemental Material files. Please contact the corresponding author directly for reuse.
