# Supplementary material for: Alternative quadruplex real-time PCR reactions for detection and discrimination of Streptococcus pneumoniae serotypes within serogroup 6
Source: Microbiol Spectr. 2026 Jul 20;14(9):e03045-25. doi: 10.1128/spectrum.03045-25 (PMC13532328; doi:10.1128/spectrum.03045-25)
Supplement: Supplementary file 1 [file spectrum.03045-25-s0001.pdf]

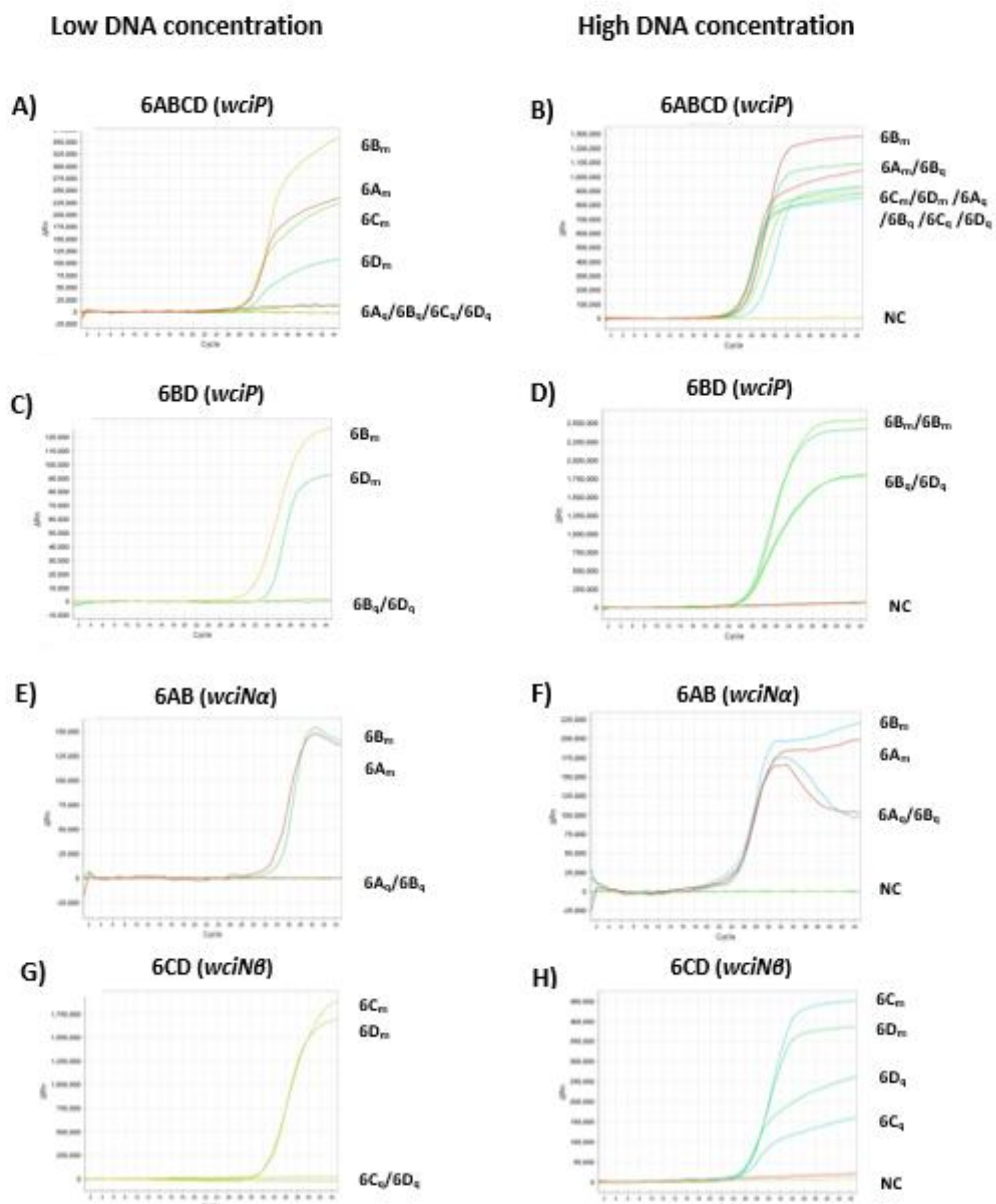

Low DNA concentration
High DNA concentration
A)
6ABCD (*wciP*)
$6B_m$
$6A_m$
$6C_m$
$6D_m$
$6A_q/6B_q/6C_q/6D_q$
B)
6ABCD (*wciP*)
$6B_m$
$6A_m/6B_q$
$6C_m/6D_m/6A_q$
$/6B_q/6C_q/6D_q$
NC
C)
6BD (*wciP*)
$6B_m$
$6D_m$
$6B_q/6D_q$
D)
6BD (*wciP*)
$6B_m/6B_m$
$6B_q/6D_q$
NC
E)
6AB (*wciNα*)
$6B_m$
$6A_m$
$6A_q/6B_q$
F)
6AB (*wciNα*)
$6B_m$
$6A_m$
$6A_q/6B_q$
NC
G)
6CD (*wciNβ*)
$6C_m$
$6D_m$
$6C_q/6D_q$
H)
6CD (*wciNβ*)
$6C_m$
$6D_m$
$6D_q$
$6C_q$
NC

Figure S1. Representative amplification plots of the monoplex and quadruplex real-time PCRs for discrimination of serotypes within serogroup 6 using low or high concentrations of DNA. Displayed are representative amplification plots for real-time PCR reactions for: A) and B) the 6ABCD target (*wciP*); C) and D) the *wciP* target for the 6BD reaction; E) and F) the *wciN*α target of the 6AB reaction; G) and H) the *wciN*β target of the 6CD reaction. DNA extracted from *S. pneumoniae* serotypes 6A, 6B, 6C, and 6D were used as template in monoplex PCR reactions (denoted $6A_m$, $6B_m$, $6C_m$ and $6D_m$) or in the quadruplex rmPCR reaction (denoted $6A_q$, $6B_q$, $6C_q$ and $6D_q$). Panels A), C), E) and G) used low concentrations of DNA for each serotype, and panels B), D) F) and H) used high concentrations of DNA. A negative control (NC) consisting of a pneumococcal serotype not detected in the reaction was used a baseline reference.

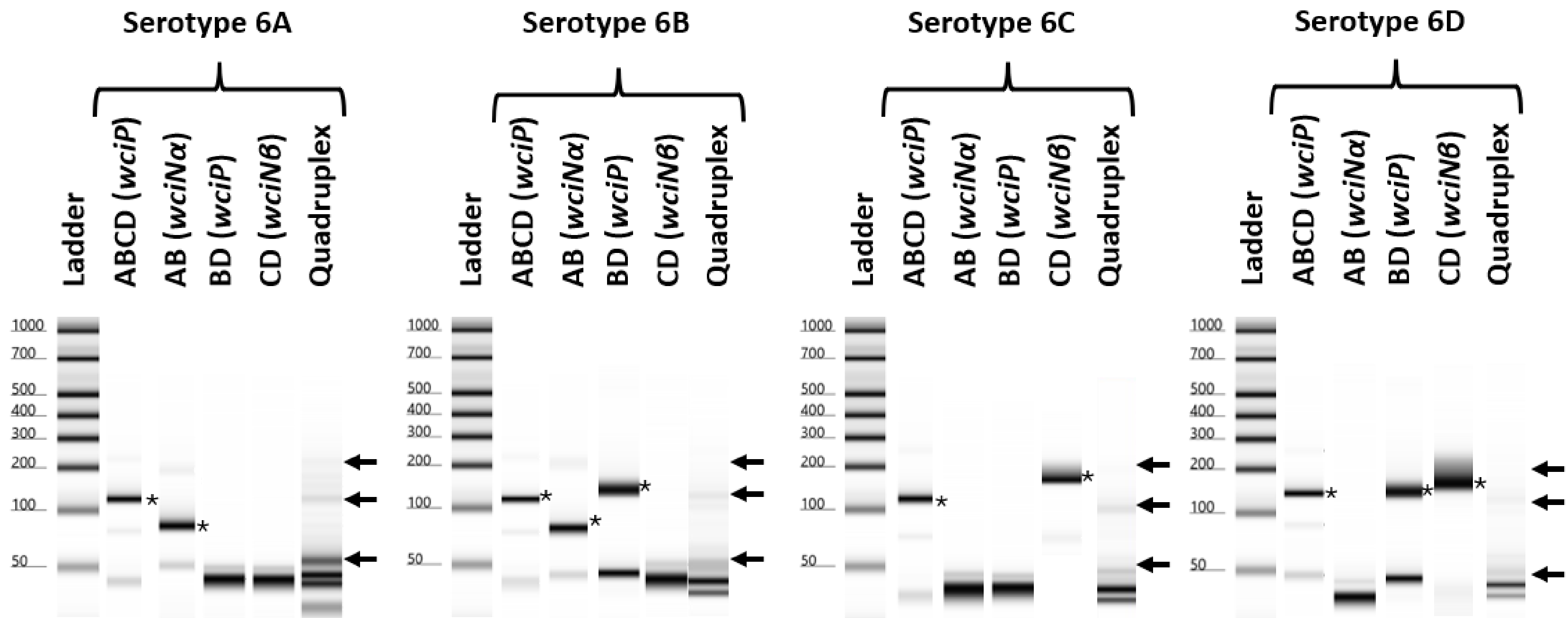

Figure S2. Analyses of amplicons generated from real-time PCR reactions using automated electrophoresis. A) Comparison between of the targets performed as monoplex PCR reactions and the quadruplex rmPCR-5 reaction for serotypes 6A, 6B, 6C, and 6D. The expected amplicons for the targets listed for each lane are denoted by asterisks (*). The size for the 6ABCD (*wciP*) target was 128 bp and was present in all serotypes tested. The 89 bp target for the 6AB reaction (*wciN*α) was present for serotypes 6A and 6B but absent for serotypes 6C and 6D. The 143 bp target of the 6BD reaction (*wciP*) was seen in serotypes 6B and 6D but is absent in serotypes 6A and 6C. The 149 bp target from reaction 6CD (*wciN*β) was seen for serotypes 6C and 6D but absent for serotypes 6A and 6B. At low concentrations of *S. pneumoniae* DNA, the rmPCR-5 quadruplex reaction for serotypes 6A, 6B, 6C, and 6D all generated a strong 57 bp artifact and occasionally weak 155 bp and 214 bp artifacts (see black arrows).

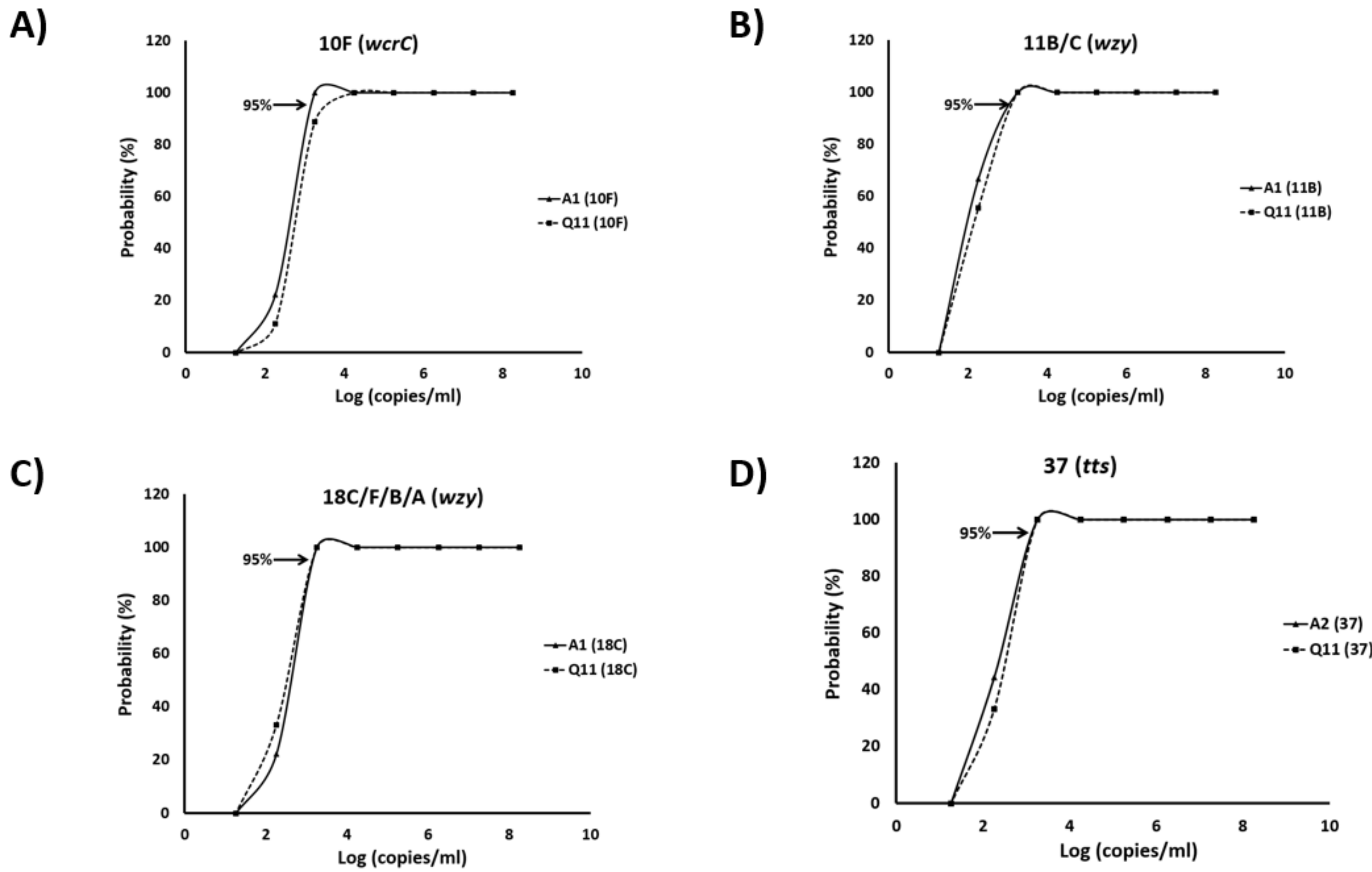

Figure S3. Probit analysis illustrating the concentration-dependent performance of traditional and alternative quadruplex rmPCR reactions for non-serogroup 6 serotypes. Ten-fold serial dilutions (n=9) were performed with normalized DNA from *S. pneumoniae* for serotypes 10F, 11B, 18C, and 37, and each dilution was subjected to the traditional quadruplex rmPCR-5 (denoted Q5) or the alternative quadruplex rmPCR-A1 and rmPCR-A2 (denoted A1 or A2) to identify the following targets: A) 10F (*wcrC*); B) 11B (*wzy*); C) 18C (*wzy*); and D) 37 (*tts*). Results are shown with dashed lines for rmPCR-5 and with solid lines for the alternative reactions rmPCR-A1 and rmPCR-A2.

Table S1. Summary of the quadruplex real-time PCR reactions target sites.

Quadruplex reaction*	Serotype(s)	Target gene (location)	Target site sequence (5′-3′)*	Size (bp)	Reference genome
1	3	*galU* (8564-8648)	**CCACTAAAGCTTTGGCAAAAGAAA**TGCTTCCAA*TTGTAGACCGCCCCACAATTCATTTTGT*CATT**TGAAGAAGCTTTACGTTCGGG**	85	CR931634.2
	22F/A	*wzy* (12961-13048)	**CTTGGGACTTCTCTATTTGTTATAGG**G*GGAGGGATAGCAATTTGGTTTCGGGA*TAAATTTTATTTT**AAAGTTGGCGGTAACTCATATT**	88	CR931681.1**
	22A	*wcwA* (8693- 8777)**	**CCCAGGACAATCACAAGAACTA**TCGGATGCGAT*TTTGGAGTTGGTTTCTGATCCAGA*AAAACGG**CTCCAATTTGGCCAAGCATCA**	85	CR931681.1
	22F	*wcwA* (6548-6629)	**CTTGTCAAGTATGCTGAGGATTTG**ATG*ACTCAACAAGCTACAGATGGACATGAAGTA*A**ATCGCATTATATCCAGGAGAAATCT**	82	LT594600.1**
2	12F/44	*wciI* (5218-5366)	**TTCGGAGGGTCCGATTATATTT**AAACAAAAACGATTTGGGATATACAAGGAACCTTTTTATATCCTGAAATTTAGGTCCATGAAAATAGGAGCACCGAA*AAATGTAGCTCCACGGAACTTGGA*TAAT**CCAGAACAGTGGATTACCAAAG**	149	CR931660.1
	23A	*wzy* (8626-8711)	**CTCCCCTCCATTACCCATTTGG**TA*TGGGAGTAGGGAGTGTGGGAGTTCTAGCT*TCAAAATTA**GGTTCACAAACAGCACTTTCTTCA**	86	CR931683.1
	35B	*wzy* (8168-8256)	**GAAAGGTATGGAGAAGTTGAGAATG***ATTCCTTACGTAGAACTGTAAGGGAAGG*TTAT**TAATAGGGTTTAATATGAATAATAGAGATGGA**	89	CR931705.1
	15A/F	*wzy* (7839-7968)	**AATTGCCTATAAACTCATTGAGATAG**TTTTACCTGATAATTTGTATACTTTTTTTAATGTTACAAAGGCATTATCATATGAGAAT*AGGACAGAGTTTGCGGG*AC**GAACAAATACTATTTCCTTCCTATGG**	130	CR931663.1

3	9L/N	wzx**<br>(11762- 11876)	**CGTGGAATTTTCTATACTGCAATAGG**GAAGTATTCGAATGTAGTTATTCAATTACTTGTAA*CAGCAATTCTTAGCCGGATTCTCTCAC***CTGTAGAATATGGTATCGTAGCAGTAG**	115	CR931647.1
	19A	wzy***<br>(9485- 9579)	**CTTAATTCGCCTAGTCTAAATACCA**AGATTA*AAGTGACGGATCGGCTCATTGATA*TCCAATTCTGGAAAATAG**CTCTTACTATTATAGTTGACCT**	95	CR931675.1
	8	_wzy_<br>(10976-11083)	**ATTCTAATTACTACATTACTGCTTTATACTA**GTATACAAATGCAAAATT*ATGTTTACTTTACGAGTTGGTTTATGTTGATT***GGTACGATTCATTATGATTTAAGAAGA**	108	CR931644.1
	16F	_wzy_<br>(12016-12214)	**TAATGTTATGACCTTGGTAATCTTCCC**CTTAATTTTTTTGATTGTTTTAGTTATATTTTATCTTTATCCTGAGAATGTTATTATAAATATGCTCTATAGCAGTTATTCTTCAACACTTAATTTGACAGATTCTTC*AGCGGTTAAGCATTTGGAAGACTTATGGCT*TCCT**CTTCTAAAAGTTATTGATTATCCTTTGGGA**	199	CR931668.1
4	33A/F/37	_wzy_<br>(11778-11882)	**GGAACTGGTTCAGCAACTATACG**CTTTGATGATTATAAGGCTGGTTT*TTTGGCATGGCAGAAAAGTCCTATTTGGGG*ATTA**GGTATTTCAGACGGTCTTAGAACC**	105**	CR931698.1
	11A/D/E	_wzy_<br>(12015-12121)	**AAATGGTTTGGATATGGTTTGTTTGG**CTTTCAATTTAAACTT*CCGTGGCAGAAATTGGGAGAAGTTGGAAT*AAATTACA**CTCATAATCAAGTTTTACAGTTAGCACT**	107	CR931653.1
	23B	_wzx_<br>(13656-13733)	**TTGAAGAAATTGCTCCAGAAACAT**A*TAGAGCTATTTATCTTTCGTGGTTTT*TG**TTAGTGGTTGAGGCTAGTCTTTTGG**	78	CR931684.1
	20	_wciL_<br>(10132-10223)	**AAAGATACTGGCTGAGGAGCTATCTATT***CAGGATAAGGTCTACTTTGTGGGAGTTC*AGTC**GAATATATCAGAATGGTTGAGTACTTTTGACT**	92	CR931679.1

5	6A/B/C/D****	*wciP*** (3408 -3535)	**GTTTGCACTAGAGTATGGGAAGG**TGT*TGTTCTGCCCTGAGCAACTGGTCTTGTATC*GAAGACATGGACATAATGTAACAACTAGTCATCATTTTAAATTATCTC**CGCTAAATGTTTTCAGAAAGGCTA**	128	AB795236.1
	6C/D	*wciN*β** (7102-7250)	**TTGGGATGATTGGTCGTATTAG**TGAACAGAAAAATCCTTTCTTTTTTGTTGAATTTGCAAAAAAATTATCAGAGATTTATAGCAATTTATATTTTGGTATTGTCGGC*GATGGCGAATTGCGTGGG*G**CGAACTGAAGAACTAATTGAAGAG**	149	EF538714.1
	6A/B	*wciN*α** (6372-6460)	**CTGATAAAGTTTCGGATAGAAATAA***AGAAAAGATAAATAGATTATCAAAACAATTTGCGCAGA***GAGAAATTAATTGGATAGAGAACGTT**	89	CR931639.1
	6B/D	*wciP*** (3322-3464)	**GCATTGTTAGAGATGGTTCCTT**CAGTTGATATTGATAAAGATTATTTATATATAGAAAA*ACTGTCTCATGATAATTATT*TTGCAAAGTTTGCACTAGAGTATGGGAAGGTGTTGTTCTGCCC**TGAGCAACTGGTCTTGTATCG**	143	AB795236.1
6	19F	*wzy* (11131-11351)	**TGAGGTTAAGATTGCTGATCG**ATTAATTGATATCCAATTTTGGAAAATTGCTCTAACTATTATAATTGATCTCTTTATTTTGTATCTTTACAGGAAGGAAATATACAACCTTGTTCTTAGTAATGGATATACGGGGTCAAATATTCAGTGGTTTTTTAGAAATGCAACGAGTTAT*GAAGGTGAATTGACAGTGCG*AA**CTTTTATTCGAGTTCTCATTCGTG**	221	CR931678.1
	31	*wzy* (9382-9496)	**AGGTTGGGACAAACCTTGC**GGTTTTACGTGGGGGGTTTGAAAAT*CCCTTAGTGACATCTGTAATGCTATCTTCT*ACATTATTATTTTTTAT**GACTATTGAGAAGGCTCTCTTACG**	115	CR931695.1
	7B/C1	*wzy* (11978-12072)	**GTGAAAAAAAGTAGTACGTTACATAG**T*AGTACGTTACATATAGGACTTATTCTTTTTTTGATTGT*AC**TGAGTAAAAACTTCTTTAATTTAGTACC**	95	CR931642.1
	7B/C2	*wchF* (7013-7095)	**TTGAGCATAACGGAGCGATA**TGTTTTAATATAGA*TGTTCCGAATATTGGTCCAGCTCGAG***CGATTGCTTATGATATAGCTGCT**	83	CR931642.1

7	10A	*wcrG* (12439-12546)	**TAGTGTCGGCAGACAAATTAT**TTAAATTCTTTGATCAGAACAAATCAAATAATTTT*TTGAGCATGGTCTCTGATGAGATTT*TGAAA**TCAAATAAAGTGTATGAGCGTG**	108	CR931649.1
	7F/A	*wcwH*** (14101-14204)	**ATGAAGGCTTTGGTTTGACAGG**AGCAGAGAGTATGGCTTGTGGC*TGTGCGTTAGTCTCAACAGCCTATAGTGGTGT*ATTT**GAATATGCAATTGATGGCGAGAAT**	104	CR931643.1
	15B/C	*wzy* (7761-7847)	**CATAGTATTTGTAGTAATGGTTCAGATT***ACTTCAATTAATAAGCGGATGATTGTAGCGT*T**TTATCCAACTATACCTCTTATATTGCT**	87	CR931664.1
	17F	*wciP* (10574-10702)	**CGGAATATCATGGAGCCTATTA**TAATTTTTGGGGGTTATTAAACGATGTTAGACAAAACGAAAATCCATTTGATTTCTATA*TGTTTGCTGATCAGGATGATATCTGG***GATGTGGACAAATTAGAACGTT**	129**	CR931670.1
8	4	*wzy* (10521-10734)	**GCTTCTGCTGTAACTGTTGTGC**CTAGTAGCATTCCATATGAATATGGGATGACATTTCTACGCACTATCCTATCAATTTTTCCTATTGGATGGTTAGTTGGTGATTTTTTTGATAAAGCATCAGCGACGGTTGTTATTAATAAATTTT*TGGGGTTACCTGTAGGCTCTTCTTTTGTGGAA*GAATTATTTT**GGAACTTTGGTTACTATGGTGGTG**	214	CR931635.1
	34	*wzy* (7683-7820)	**CGTGGAAGTTTCTCGCAAATAA**TTAATAACTATGACCAATTAGTAAAGTTT*TTTACTGAAGACTTAGTCGGATTGGG*GGTCAGGTCTCCGTTTTTTTATAGAGTGTTTAATA**GCTTCATATCTCCTATTTCTTACGTG**	138	CR931703.1
	35F/47F	*wzy* (7694-7838)	**GTGGTCGTATATACTTGATGAATAAATCG**TTGAATTTGTATGGTTTT*GGATTATTCGGACGACCAGTTGAATGGA*ATGGAAATGGTCTGACCATTGAAGGAGTTAGAAACTATCA**GACCTATCTGTATGTTGATAATTTGTATGT**	145**	CR931707.1
	38	*wcyV* (18262-18344)	**TGATGAGCTTCCAAATTCTTTT**G*TGATGAAAACCAATCATGATAGTGGCAGTATTA*TC**ATTGTAAAAAACAAAGAAGACTTGA**	83	CR931710.1

9	21	*wzy* (12355-12441)	**GGTTTAAATATCGCTCCGGGTAT**AATTGAAGAAAGAGG*TGTGAATTGGACACGTTATGGAGC*GG**TTCGTCTACAAGCCCTTTTTTTG**	87	CR931680.1
	13	*wzy* (13163-13298)	**AGACTACCATTTTTTGATCAGTTAGATT**TAATATTAACA*GGTAGATTACGACTTGGAAGTGCTGCTT*TAATAATTTTCACATAACATTTTTTGGACAAAAAATAA**GATGGATTTATGAACAAAATATGTTTTCTG**	136	CR931661.1
	24F/A/B	*wzy* (12323-12415)	**GGAGCGGGATATATTTCTTCTAGTC**CATATTTTGAAACTTAT*TCTATTGTTACTGGTCCATTAGGACGT*T**ATTATCCAAGCGATGTAGGTTTG**	93	CR931688.1
	9V/A	*wzx* (11767-11920)	**AGGTATCCTATATACTGCTTTAGG**TAAGTATTCTAATGTTATAATTCAATTATTTGTAACGGCGGTATTAAGTAGAATATTAACACCTGAAGAGTATGGCGTTGT*AGCGGTTGTCAATGTGT*TTTTAGTATT**CTTTCAGATATTGGCAGATTCG**	154	CR931648.1
10	1	*wzy* (9875-10035)	**TTTCATCCCTATGTGTGGTATAG**TAGCTTCTTTACTATTTATTCTATTTCTTACCCGCTACTCTATAGAATGGAGTATATAAACTATGGTTATACAAAAGATTTGATTTTCATGCA*ATGGCTGGCTTTGGCA*ACTTT**GTTGTTAACTCTACCTTCTAAAGC**	161	CR931632.1
	14	*wzy**** (7915-8007)	**GCTTTAGAGTGTATGAGGAATCC**CTAAAAGCTATT*AATGGAATGGAAATGTTACTTGGCG*CAGGTGTCAGA**ATTCCCTCTACAGTAGATATAT**	93	CR931662.1
	23F	*wzy* (9049-9274)	**GACAGCAACGACAATAGTCATCTC**TTTACTGGCGTTAACATTTTTTTTCAAACATATAATTAATAATAGTGAATCATATAGCCATCGAGTGTTAGGTGTTGTGAATTTTTTTAAATATTATGAATCAGATAGGTTTCATTTGTTTTTTGGGGATGCTGAATTAGC*CTTTGGAAATACGACGAAGGGTTATGGACACAAT*ATTA**GAAGTGTGTTAGGTTGGGATGGA**	226	CR931685.1
	28A/F	*wzy* (10658-10753)	**TTTAGTTCGTGGAGGTAGACT***ACCAATTTCAATTCCAGGAGCGAA*ATATTATTTTACTTTATTAATGGTGG**GGCTATTTATAGGTATTGGGAATGT**	96	CR931692.1

11	37****	*tts* (2220-2310)	**GATGCCAGCATTTATTCACACC**GTT*AGAGACGCCAGTGAGTATCAAGGAGT*ATTGAGGCCTTTG**GTATTGGTATTAGTCGTATTGAATGCC**	91	AJ131985.1
	11B/C	*wzy* (12516-12653)	**CCGCTATCAAATTTGGCGTATTG**CTTATCAAAAATTTTTAGATTCAAAAATATATGGCTATGGAGTTTTTAGCTTTCATTTCAATCT*TCCGTGGCAAGATTCTGGTGCTAA*GG**GGATCAACTATGCTCATAATCAGCT**	138**	CR931655.1
	10F	*wcrC* (8881-9026)	**ATAAATTATGATGTTAATCCTAATGTGAC**ATTAATAAATTTTCACAAGGGTGATCTTAGAATTAGAACTGCTACAT*TAAAATTAACGGAGAAATTAAGGGATTATATTAAAAGAGAAA***AAATTAAAGTGATTTTTTCTATAACCCC**	146	CR931652.1
	18C/F/B/A	*wzy* (12934-13081)	**TCGATGGCTAGAACAGATTTATGG***AAGGGAGTTGAATCAACCTATAATTTCGCCCC*TATATTTATGGGGAGAGGGATAGGGTTTGTAACAAAATGGATGGATAATAATTGGATGACTTTGAATAT**CAATGGTCTTACAGGGACAATGG**	148	CR931673.1
12	5	*wzy* (7001-7082)	**CATGATTTATGCCCTCTTGCAA**CGT*TCTTCTTCTCATCGTTTCCGCATGCTTTTT***TTAGCCCTTGCTTTTTCTTATACTGTC**	82	CR931637.1
	39	*wzy* (11321-11431)	**TGCGCTAAGGTATATTCCGTATTT**TAC*TGATGATGGAGCCTATCATTATAAAGCAGC*TTATTTATTTCAGTTTTATGATAATGTTTTT**GATTGGTTTGGGAACTTGATGTC**	111	CR931711.1
	35A	*wzy* (7389-7467)	**TTCCTGATTATGTTGAGATTTGGC**CT*ACCAGAGTTAGACACTATCTTGGTTTCC***GATATTCATTACTTCCATCAACGCT**	79	CR931704.1
	2	wzy (10342-10452)	**TGTTATCCCATATAAGAACCGAGTGT**TAAATTTAGTTTATAAAACACTTA*TTGCAATTTCAATTTTTTTGCCCCAATCTC*GTATAGT**TTGGATAGCTTTTGGGGTAATTTT**	111	CR931633.1

*Based on Velusamy et al. [22]. **Corrected from errors noted in Velusamy et al. [22]. ***Compared to the traditional quadruplex PCRs [22], forward primers for serotypes 14 and 19A contain at the 5' end an additional 5- or 7-bp, respectively (underlined). ****In the alternative quadruplex reactions, primers and probes for target 6ABCD from reaction 5 were exchanged those of serotype 37 from reaction 11, and vice versa.

Table S2. Non-pneumococcal streptococci and Gram-positive cocci used in the specificity analysis.

Organism	ATCC No.	Lancefield group
Streptococcus pyogenes	19615	A
Streptococcus agalactiae	12386	B
Streptococcus dysgalactiae subsp. *equisimilis*	12388	C
Streptococcus equi subsp. *zooepidemicus*	700400	C
Streptococcus equi subsp. equi	33398	C
Streptococcus gallolyticus subsp*. gallolyticus*	43143	D
Streptococcus gallolyticus subsp. *pasteurianus*	700338	D
Streptococcus porcinus	35650	E
Streptococcus sp. strain [Lancfield H59]	9328	F
Streptococcus anginosus	33397	G
Streptococcus dysgalactiae subsp. *equisimilis*	12394	G
Streptococcus sp. strain D167B [SHC80]	9933	L
Streptococcus sp. strain D168BX	9935	M
Streptococcus sp. strain C559 [O.J]	9936	N
Streptococcus sp. *mitis* strain B357	11843	O
Streptococcus suis	43765	R
Streptococcus salivarius subsp. t*hermophilus*	19258	
Streptococcus pseudopneumoniae	BAA-960	
Streptococcus infantarius subsp. *infantarius*	BAA-102	

Streptococcus mitis	49456	
Streptococcus oralis	35037	
Streptococcus gordonii	12396	
Streptococcus sanguinis	49298	
Streptococcus mutans	35668	
Streptococcus salivarius subsp. *salivarius*	7073	
Streptococcus constellatus subsp. *constellatus*	27513	
Streptococcus intermedius	27335	
Enterococcus faecium	19434	D
Enterococcus faecalis	19433	D
Abiotrophia defective	49176	
Granulicatella adjacens	49175	
Staphylococcus aureus	25923	
